# A Spectrum of Chemoport-Associated Complications and Their Management in Cancer Patients

**DOI:** 10.7759/cureus.58052

**Published:** 2024-04-11

**Authors:** Vikas Sharma, Arun Pandey

**Affiliations:** 1 Surgical Oncology, Dr. Ram Manohar Lohia Institute of Medical Sciences, Lucknow, IND; 2 Surgical Oncology, Geetanjali Medical College and Hospital, Udaipur, IND

**Keywords:** infection, chemotherapy, complication, chemoport, vascular access device

## Abstract

Introduction: Chemotherapy is part and parcel of the multimodality approach to cancer treatment. Chemoports are frequently used to administer chemotherapy, preventing complications associated with the use of peripheral lines. However, chemoports have their own set of complications and can be very debilitating at times. Accurate knowledge and correct technique can help prevent and manage these complications properly.

Methods: We retrospectively analyzed all patients who underwent chemoport insertion for chemotherapy infusion over three years between July 2020 and June 2023. The patient's profile, type of cancer, the technique of chemoport insertion, complications related to chemoport, and its management were recorded retrospectively from patient records.

Results: The total number of patients in our study was 119. The age group of patients ranged from 13 years to 76 years. Of the 119 patients, 55 had breast cancer, 23 had ovarian cancers, 29 had GI cancers including gastroesophageal junction (GEJ)/ stomach/periampullary/colorectal, and 12 had leukemias. The most common intraoperative complication was catheter tip malposition (9.2%). The most common postoperative complications were infection (7.5%), followed by drug extravasation (5.0%), thrombosis (3.3%), wound dehiscence (2.5%), and skin necrosis (0.8%) in decreasing order of frequency. Serious complications such as hemothorax, pneumothorax, air emboli, brachial plexus injury, and pericardial tamponade, commonly reported in the literature, were not seen in any of our cases.

Conclusion: Totally implanted venous access devices (TIVAD)/chemoports are indispensable in the management of cancer patients, especially in patients requiring long duration of infusion and prolonged treatment. Although chemoports are associated with a spectrum of complications, proper technique of implantation and use makes it a safe and reliable tool.

## Introduction

Chemotherapy is part and parcel of the multimodality approach to cancer treatment. Other than the usual and specific side effects of various chemotherapeutic agents, infusion-related complications are common to every chemotherapeutic regime. Thrombophlebitis, skin necrosis, and cellulitis are common occurrences when peripheral veins are used for chemotherapy infusion, especially in patients requiring chemotherapy for long durations. The totally implantable devices were introduced in the 1980s and since then chemoports are frequently used to administer chemotherapy, preventing complications associated with the use of peripheral lines [1]. However, chemoports have their own set of complications and can be very debilitating at times. Minor complications are events, which do not require additional surgical or interventional therapy or medical therapy >24 hours, whereas major complications require surgery/intervention, prolonged medical therapy, a hospital stay >24 hours, or even result in death. Hemothorax and pneumothorax are the most likely major complications, based on the severity. The overall complication rate has been reported to be 7.2-12.5%, with port system infection being the most common [2,3]. With an incidence of 5-18%, catheter-related thrombosis is also relatively common and does not necessarily require catheter explantation. Accurate knowledge and correct technique can help prevent and manage these complications properly. Here we share our experience of chemoport-related complications and their management.

## Materials and methods

We retrospectively analyzed all patients who underwent chemoport insertion for chemotherapy infusion over three years between July 2020 and June 2023. Patients' profiles, type of cancer, the technique of chemoport insertion, and complications related to chemoport and its management were recorded retrospectively from patients' records.

The technique of chemoport insertion

Our preferred site of chemoport insertion is the right subclavian vein. The left subclavian vein is chosen in cases of carcinoma right breast. In all patients, chemoport insertion is done under local anesthesia under strict aseptic precautions. The catheter is tunneled subcutaneously to the pectoral region. The port chamber is connected to the catheter and placed under the thick skin subcutaneous tissue flap in the pectoral region. This requires short tunneling and avoids passing the catheter across the clavicle as compared to when the internal jugular vein (IJV) is used for chemoport access. The catheter tip is placed at the junction of the superior vena cava (SVC) and the right atrium. Proper catheter placement is checked using an intraoperative C-arm. We prefer chemoport use usually after 48-72 hours of insertion. We use chemoport exclusively for chemotherapy infusion only.

## Results

The total number of patients in our study was 119. The age group of patients ranged from 13 years to 76 years. Of the 119 patients, 55 had breast cancer, 23 had ovarian cancers, 29 had GI cancers including gastroesophageal junction (GEJ)/ stomach/periampullary/colorectal, and 12 had leukemias.

The overall incidence of all complications combined was 31.9% in our study. Complications seen are divided into intraoperative and postoperative. Postoperative complications were further divided into early postoperative (<30 days) and late postoperative (>30 days). A list of all complications encountered are shown in Table 1.

**Table 1:** Complications encountered and their incidence IJV: internal jugular vein

Intraoperative complications	
Catheter tip malposition (ipsilateral IJV, contralateral IJV, cardiac)	11 patients (9.2%)
Subclavian artery puncture	3 patients (2.5%)
Postoperative complications	
Thrombosis	4 patients (3.3%)
Infection (early/delayed)	9 patients (7.5%)
Wound dehiscence	3 patients (2.5%)
Reservoir rotation	1 patient (0.8%)
Drug extravasation	6 patients (5.04%)
Skin necrosis and reservoir exposure	1 patient (0.8%)

**Figure 1:**
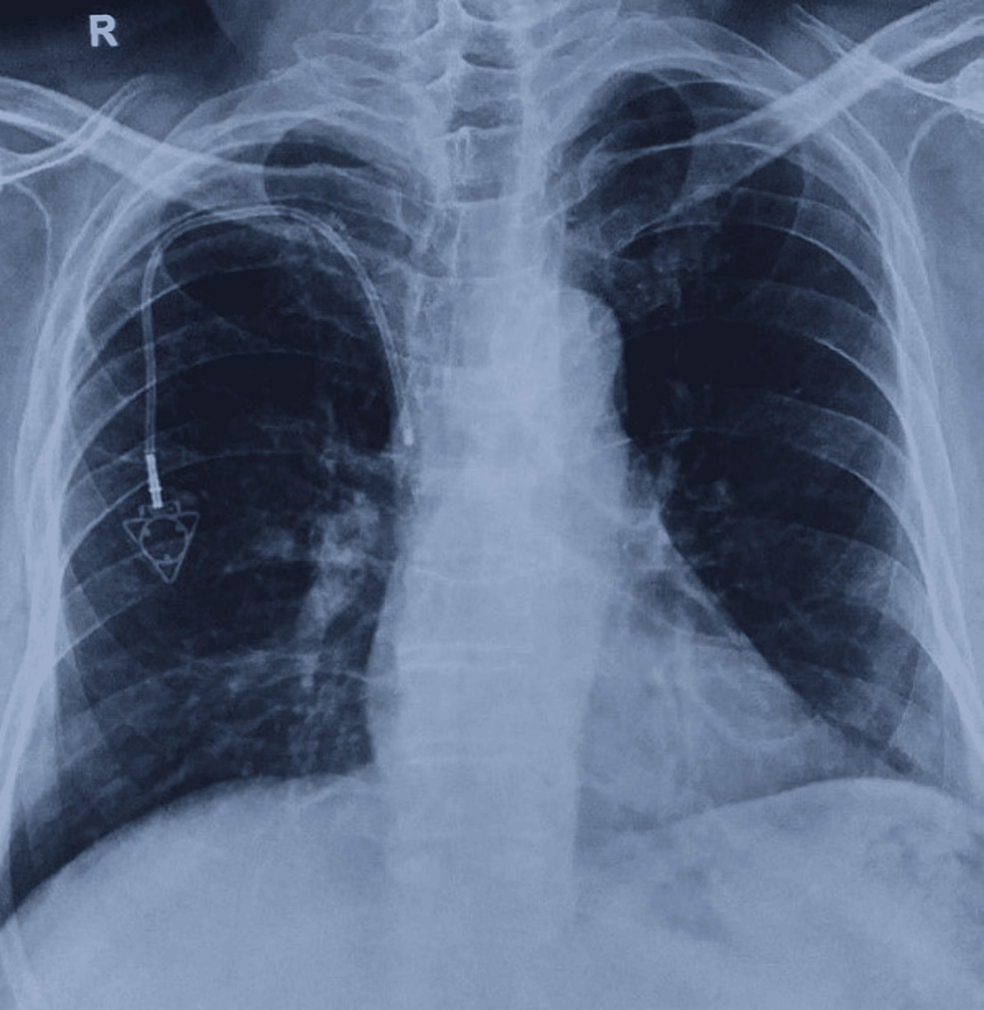
The normal position of implanted chemoport through the right subclavian vein. The tip can be seen in SVC at the junction with the right atrium. SVC: superior vena cava

**Figure 2:**
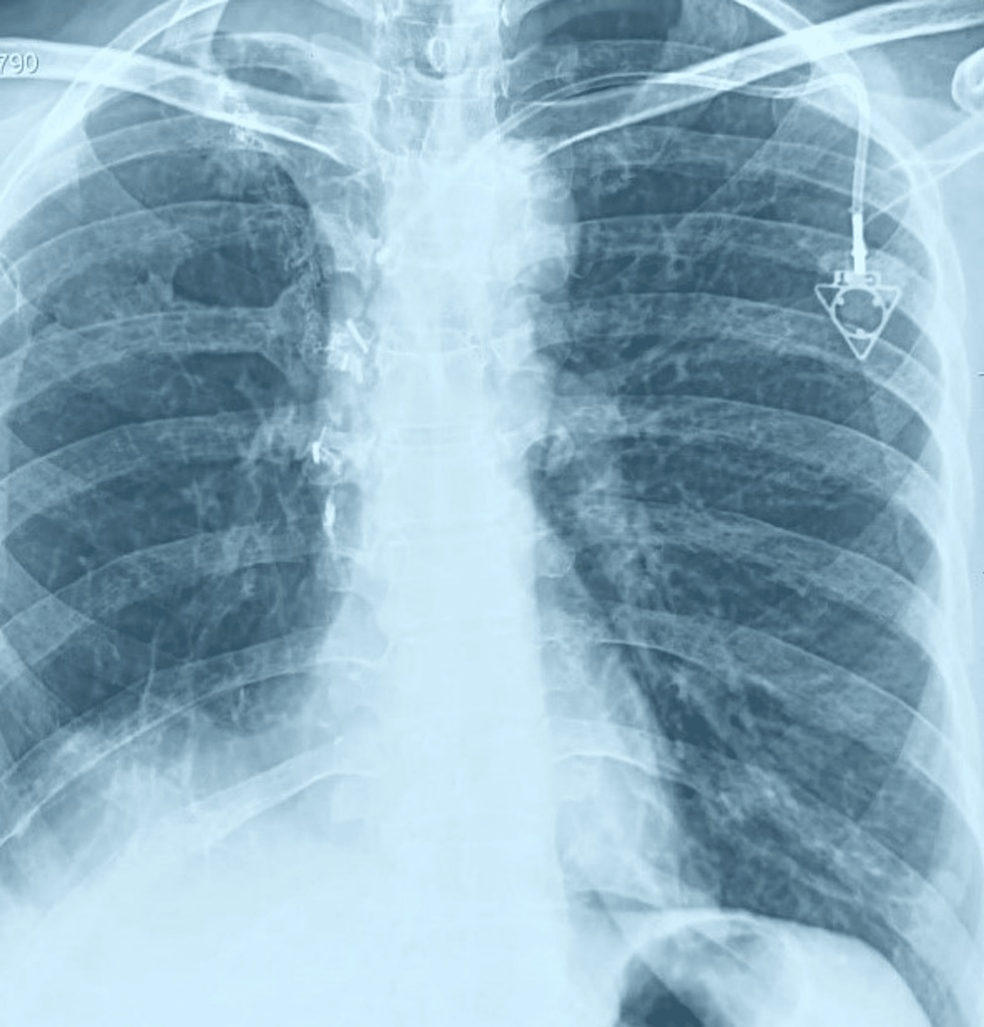
The normal position of implanted chemoport through left subclavian vein in a case of carcinoma right breast.

**Figure 3:**
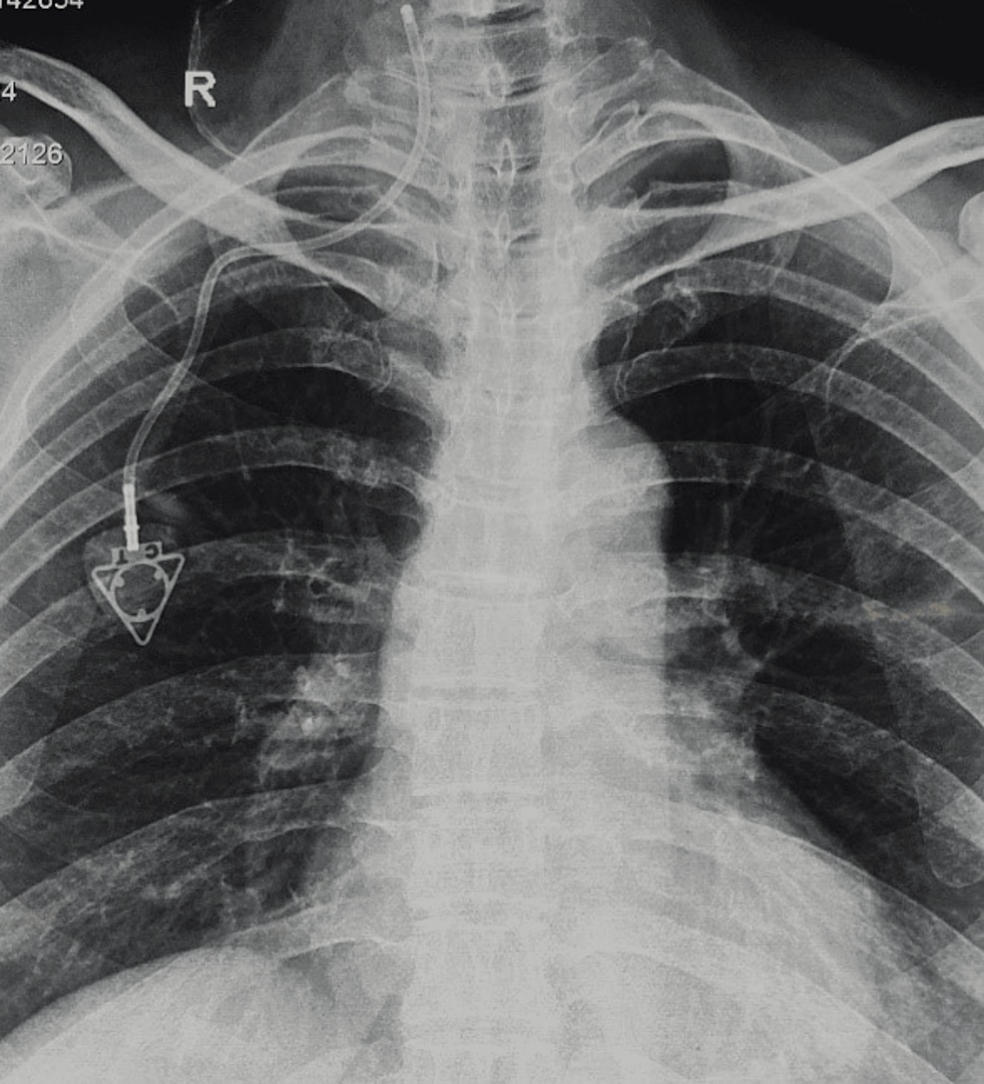
The catheter was seen malpositioned into the right IJV. IJV: internal jugular vein

The most common intraoperative complication was catheter tip malposition (9.2%). Normal position is shown in Figures 1-2, where the tip can be seen positioned at the junction of SVC and right atrium. Catheter tip malposition into right IJV can be seen in Figure 3. Subclavian artery puncture was encountered in 2.5% of patients.

**Figure 4:**
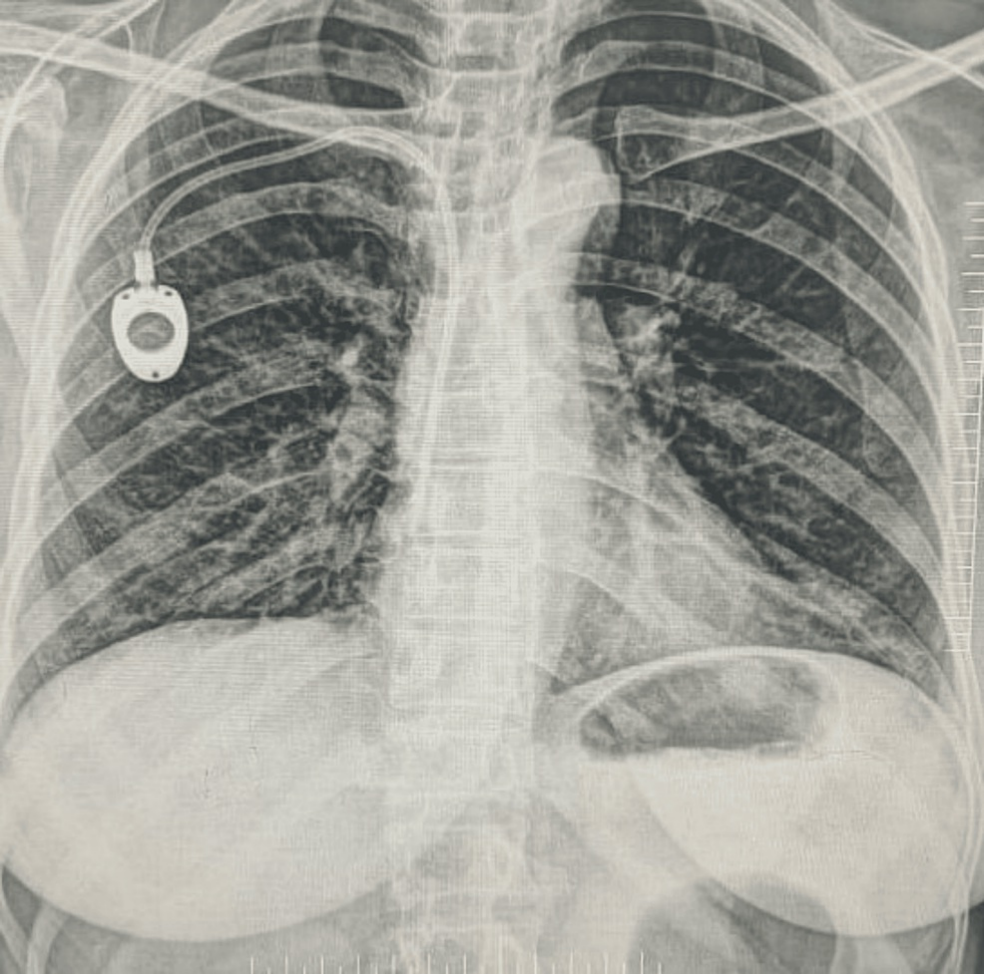
Reservoir rotation results in the inability to puncture the chamber. The reservoir can be seen flipped 180 degrees.

Most common postoperative complications were infection (7.5%), followed by drug extravasation (5.0%), thrombosis (3.3%), wound dehiscence (2.5%), and skin necrosis (0.8%) in decreasing order of frequency. One patient developed reservoir rotation as can be seen in Figure 4.

## Discussion

Chemotherapy administration very commonly causes local toxicity secondary to peripheral venous administration and extravasation of these drugs. The inclusion of central venous devices has significantly reduced complication rates associated with chemotherapy administration. Of these devices, totally implantable venous access devices (TIVAD) have the practical benefit of being logistically simple to manage. These devices, however, have their own set of problems associated with them.

Transient intraoperative cardiac arrhythmias were seen in 30 (25.21%) patients in our study. Cardiac arrhythmias are usually caused by mechanical irritation of the endocardium while entering the right atrium. All cases in our study were clinically insignificant rhythm abnormalities that were reverted back to normal rhythm simply by withdrawing the guidewire/catheter, although more serious clinical situations like asystole and complete heart block have also been reported in the literature [4-6]. It is important to monitor the ECG and movement of the guidewire and catheter during the procedure. Particular care should be taken in patients with preexisting cardiac abnormalities [7].

Catheter tip malposition was detected in 11 patients in our study. Ideal catheter tip positioning is near the SVC cardiac junction. In four patients it went into ipsilateral IJV, in one patient into contralateral IJV, and in six patients into the right atrium. Routine use of fluoroscopy reduces the incidence of this complication and also actively helps in correcting the position during the procedure itself. In all patients, malposition was detected on intraoperative fluoroscopy and was repositioned immediately.

The inadvertent arterial puncture has been reported in the literature in the range of 0.5-3% for subclavian vein and IJV catheterization respectively [8]. In our study, subclavian artery puncture was reported in a total of three cases (2.5%). Subclavian artery puncture is less common but can be more problematic due to its deep location and difficult compressibility. It was detected immediately and the needle was withdrawn. Pressure was applied for five minutes at the site of the puncture to prevent bleeding and hematoma formation.

The overall incidence of intraoperative and postoperative complications of chemoport insertion ranges from 1.4% to 11.1% and from 6.5% to 17.1% in the literature [9,10]. In our study, this incidence was seen to be 11.76% (other than cardiac arrhythmias) and 24.36% respectively. While the overall complication rate was higher in our study, most complications were inconsequential and clinically insignificant.

Other early and serious complications such as hemothorax, pneumothorax, air emboli, brachial plexus injury, and pericardial tamponade, commonly reported in literature, were not seen in any of our cases [11-15].

The most common complication in our study was chemoport-related infections which were found in 11 patients (7.5%). In the reported literature, it has been the most common cause of the need for port removal before treatment completion and ranges from 5.6% to 8% [16-18]. Most of these infections were seen more than 30 days after the procedure. In four patients, the site of infection was a port pocket/tunnel and presented with local swelling, pain, redness tenderness, and pus discharge in one of them. The rest of the seven patients had catheter-related bloodstream infections (CRBSI) and presented with hypotension, chills, and signs of sepsis of sudden onset after catheter use. Three patients presented early (<30 days) and eight had delayed onset(>30 days) after chemoport insertion. The most common organisms isolated were *Staphylococcus aureus*, *Klebsiella pneumoniae*, *Pseudomonas aeruginosa*, and *Enterobacter *spp. Other than these agents, coagulase-negative staphylococci and candida species are also commonly found in these infections [19]. Most of the patients were managed by antibiotic therapy after obtaining pus/blood cultures. Three patients required port removal because of features of severe sepsis. The inclusion of strict sterile protocols during the insertion of port and venous access should be followed to prevent these infections.

Catheter blockage due to thrombosis was found in four patients (3.3%) and was detected using Doppler ultrasound, which correlates well with the range described in the literature (1-5%) [20]. Regular flushing of the port reduces the risk of thrombosis. Conventionally, heparin has been used for flushing the ports but some studies including one randomized trial have shown that normal saline is non-inferior to the conventional method [21,22]. Treatment with low-molecular-weight heparin (LMWH) alone or followed by warfarin for around three to six months duration is usually recommended. Catheter removal is required in patients with sepsis, a concomitant deep vein thrombosis (DVT), or if the port is no longer needed. A short course of anticoagulation is required for three to five days before port removal for thrombosis [23]. In two patients it was managed using LMWH and the prophylactic dose was continued till the catheter was in place. In one case the catheter could not be used because of associated sepsis and was removed after five days of anticoagulant therapy. In one patient catheter was removed because of the completion of chemotherapy and anticoagulation therapy was given for three months.

Wound dehiscence was seen in three of our cases. In all three cases, wound gape occurred at the time of suture removal. Skin necrosis with implant exposure was seen in one patient. Wound healing in these patients may be delayed due to poor nutrition and an immunocompromised state. Avoiding implantation under irradiated skin and tension at the suture line reduces the risk of dehiscence of the suture line. A recent use of bevacizumab has also been found to be associated with an increased incidence of this complication, and the risk is highest if the interval is less than 14 days [24,25]. Extrusion of the port due to ulceration and necrosis of the overlying skin usually necessitates removal of the port; wound gapes at the suture line however may be managed with secondary suturing [24,25].

Flipping of ports in our study occurred in one patient and was managed with re-exploration and positioning. It is usually caused by large pocket size and inadequate fixation of the port in the pocket.

Local extravasation of the drug has been reported in 0.1-6% of cases in the literature, it was however seen in 5.0% in our study [24-27] and led to local inflammatory reaction necessitating discontinuation of chemotherapy through chempoport for a couple of cycles in few of the patients. In three cases the extravasated drug was paclitaxel, in one adriamycin and in two cases it was oxaliplatin. In only one case the extravasation was major and required chemoport removal owing to overlying skin necrosis and port exposure. In rest five cases it was very minimal extravasation and was managed by antiinflammatory drugs. The extravasation may be due to catheter fracture, disconnection, or leakage from the port diaphragm. Using coring needles or leaving needles in situ for a long time may increase the risk of a diaphragm leak. The patient usually presents with local swelling and inflammation with an inability to aspirate blood [28]. Flushing and aspiration of the port should precede any drug administration to rule out local swelling, pain, or inability to aspirate back. An abnormal finding should dictate an immediate stopping of the use of the port for any infusion. The extravasated fluid should be removed as much as possible. In our study, this was usually sufficient along with anti-inflammatory drugs to settle down the local reaction. The use of local or systemic steroids should be avoided [23]. One of the patients developed skin necrosis, probably owing to a thin flap, leading to port reservoir exposure. This patient was managed by port removal, conservative wound management, and reinsertion of chemoport on the opposite side.

The strength of our study is that we could manage to report the various spectrum of complications and how to adequately manage them and how a good technique could prevent life-threatening complications (like hemothorax, pneumothorax, air embolism, etc.) associated with chemoport insertion, as none were seen in our study. The main limitation of our study is that it is retrospective in nature.

## Conclusions

TIVAD/chemoports are indispensable in the management of cancer patients, especially in patients requiring long duration of infusion and prolonged treatment. Although chemoports are associated with a spectrum of complications, proper technique of implantation and use makes it a safe and reliable tool. Although the reported incidence of complications is higher in our cases, most were minor and managed conservatively. Major life-threatening complications like pneumothorax, hemothorax, and air embolism were not seen in our study, showcasing the importance of proper technique of insertion.
